# Children’s Comprehension of Narrative and Expository Texts: The Contributions of Word Decoding and Knowledge Integration Skills Vary Within and Between Text Types

**DOI:** 10.11114/jets.v10i4.5465

**Published:** 2022-06-07

**Authors:** Brenda Hannon

**Affiliations:** Dr. Brenda Hannon, Texas A&M University – Kingsville, Kingsville, Texas, USA.

**Keywords:** narrative, expository, component skills, beginning readers, reading

## Abstract

This study examined the concurrent (mean age: 5.96 years) and longitudinal (mean age: 5.96 years) contributions that multiple component skills of reading made to 155 beginning readers’ comprehension scores for narrative and expository texts. The component skills included: word decoding, text memory, knowledge integration, and working memory. For narrative texts, word decoding was one of the best predictors of comprehension scores both concurrently and longitudinally; although longitudinally, the predictive strength of word decoding was equivalent to knowledge integration. In contrast, for expository texts, knowledge integration was a much better predictor of comprehension scores than any other component skill, including word decoding. When the contributions of the component skills were considered between text types, word decoding was a better predictor of comprehension scores for narrative texts than expository texts, whereas knowledge integration was a better predictor of comprehension scores for expository texts than narrative texts. Taken as a whole, these findings suggest that the contributions that component skills make to the comprehension scores of beginning readers vary as a function of text type, and that the common assumption that word decoding is the most important skill for beginning readers may be limited to just the comprehension scores of narrative texts.

## 1. Introduction

In elementary grades, children encounter a range of tasks that require reading and comprehending texts (Eason, Goldberg, Young, Geist, & Cutting, 2012). In earlier grades, the texts that children encounter most frequently are narrative, a type of text that typically conveys information about fictional characters and plots using everyday language (Graesser, McNamara, & Kulikowich, 2011). As children progress through elementary grades, the dominance and importance of narrative texts shift to expository texts, a type of text that typically conveys facts about specific topics utilizing technical language (Graesser et al., 2011). This shift most likely occurs because of increased emphasis on independent reading as the primary source of knowledge in later elementary grades (Kraal, Koornneef, Saab, & van den Broek, 2017).

Despite the prevalence/importance of both types of texts, we still, surprisingly, have limited knowledge about the relative contributions that the component skills of reading comprehension make to the comprehension scores of narrative and expository texts (Francis, Kulesz, & Benoit, 2008), especially when the population of interest has minimum reading experience and the component skills are assessed before a child begins to read. In this study, we assessed, concurrently and longitudinally, component skills that are highly predictive of reading comprehension in order to determine their relative contributions to beginning readers’ comprehension scores on narrative and expository texts. More specifically, we used structural equation models (i.e., SEMs) to examine the relative contributions that some important component skills make to comprehension of: (i) just narrative texts (i.e., within-text contributions), (ii) just expository texts (i.e., within-text contributions), and (iii) narrative versus expository texts (i.e., between-text contributions).

### 1.1 Component Skills

Most researchers agree that successful reading comprehension requires the formation of a coherent mental representation of a text (Graesser, Singer, & Trabasso, 1994). However, a coherent mental representation is not simply a composite of text-based facts or even connected text-based facts (Graesser et al., 1994). Rather, a mental representation is the synthesis of two types of text-based information, explicit and implicit, and two sources of knowledge, the text and the reader (Hannon, 2012; Hannon, Teague, Ehlringer, & Johnson, 2016; Kintsch, 1998).

A mental representation of a text also requires the co-ordination and execution of a number of component skills (e.g., Cain, Oakhill, & Bryant, 2004; Silva & Cain, 2014) and cognitive resources (Hannon, 2012; Hannon & Daneman, 2001). Good word decoding skills, which are essential for successful reading comprehension (Best, Floyd, & McNamara, 2008), identify and sound out words as well as access the meanings of words (Hannon, 2018). Word decoding skills are especially important for young children who are just learning how to read and comprehend texts. Indeed, research suggests word decoding skills account for as much as 55% of the variance in children’s reading comprehension scores (Garcia & Cain, 2014).

Text memory and knowledge integration skills, which extract information from a text (i.e., text memory) and integrate this text-based information with a reader’s prior knowledge (i.e., knowledge integration), are also essential for successful reading comprehension. Knowledge integration skills, in particular, are highly predictive of early reading comprehension (e.g., Silva & Cain, 2014), accounting for as much as 26% of the variance in the reading comprehension scores of beginning readers (e.g., Cain et al., 2004). In addition, working memory, a cognitive resource that is shared by a number of component skills of reading comprehension (Daneman & Merikle, 1996; Hannon & Daneman, 2001), is especially important for forming a text representation because component skills use this resource to integrate text-based information with information from a reader’s prior knowledge (McNamara & Magliano, 2009). Working memory capacities are also predictive of the comprehension scores of beginning readers both longitudinally (e.g., Silva & Cain, 2014) and concurrently (e.g., Cain et al., 2004). However, the predictive power of working memory diminishes when measures of inferential and knowledge integration skills are included in the analysis (e.g., Hannon & Frias, 2012).

### 1.2 Narrative and Expository Texts

Narrative and expository texts differ in multiple ways. Narrative texts typically convey information about fictional characters and plots utilizing everyday language and story structures that follow temporal sequences (Graesser et al., 2011). Biographies, which typically follow temporal sequences, are often narrative in nature, although they do not include fictitious characters and plots. Expository texts, on the other hand, convey facts about specific topics utilizing technical language and global structures that often fail to follow specific timelines or structures. Many scientific texts are expository in nature.

It is generally believed that expository texts are more difficult to comprehend than narrative texts (e.g., Best et al., 2008; Diakidoy, Stylianou, Karefillidou, & Papageorgiou, 2005; Kraal, van den Broek, Koornneef, Ganushchak, & Saab, 2019), possibly because: (i) readers are less familiar with the global structures and reading comprehension goals of expository texts (Kraal et al., 2019) and/or (ii) expository texts tap inferential skills to a greater extent than narrative texts (Kaakinen et al., 2003). However, findings concerning this point are mixed. Eason et al. (2012), for example, showed that the comprehension scores of 10 to 14-year old readers were equivalent on narrative and expository texts.

When the component skills of reading comprehension are considered, most studies have examined the contributions of these component skills using just narrative texts, just expository texts, or both narrative and expository texts without differentiating the two text types apart (Eason et al., 2012). Further, few studies have examined the contributions of component skills to expository texts in early elementary grades (Kraal et al., 2017). Nevertheless, the few studies that have examined the contributions of component skills to narrative versus expository texts suggest both similarities and differences in the size of the contributions that these component skills make to these two text types.

Studies consistently show that word decoding is highly predictive of both narrative (Best et al., 2008) and expository texts (Garcia & Cain, 2014). It is also generally accepted that word decoding is a better predictor of narrative than expository texts (Garcia & Cain, 2014), *r = .63* versus *r* = .30 respectively (Garcia & Cain, 2014). The only exception to this generality is Eason et al. (2012), who showed that word decoding is equally predictive of narrative and expository texts, *r* = .46 versus *r* = .54, z < 1.00.

Nevertheless, it is unclear whether word decoding is the best single predictor of narrative texts. Best et al. (2008), for example, showed that word decoding was more predictive of narrative texts than world or prior knowledge. On the other hand, Eason et al. (2012) showed that vocabulary was a significantly better predictor of narrative texts than *any* other predictor including word decoding, *min z* = −1.72, *p* < .04. Given that these studies used different predictors (i.e., word decoding and prior knowledge versus word decoding, vocabulary, syntactic skills, inferential skills, planning, and organizational skills) as well as different word decoding measures (i.e., identification versus fluency), clearly more research is needed in order to answer this question.

Finally, few studies have examined the relative contributions of inferential and knowledge integration skills to narrative versus expository texts. However, it is generally believed that inferential and knowledge integration skills are predictive of both types of texts (Kraal et al., 2019), and some researchers have gone as far as to suggest that expository texts require stronger inferential and knowledge integration skills (e.g., Kraal et al., 2019). Nevertheless, to date there is no direct evidence suggesting inferential and knowledge integration skills are more predictive of expository than narrative texts. In fact, to the best of our knowledge, the only study that has examined the relative contributions of inferential and knowledge integration skills to narrative versus expository texts reported equivalent contributions, *r* = .37 versus *r* = .40 respectively, *z* < 1.00 (Eason et al., 2012).

### 1.3 Summary and Current Study

In summary, although a few studies have examined the contributions that component skills make to narrative and expository texts, we still have limited knowledge about their contributions. This lack of knowledge is problematic for two reasons. From a practical perspective, it leaves teachers and reading professionals uninformed about the important component skills for specific types of texts, and consequently, limits their ability to customize reading interventions as a function of text type for poor readers. From a theoretical perspective, it limits our knowledge about reading comprehension. For example, are inferential and knowledge integration skills more important for expository texts than narrative texts?

In addition, because many studies examining differences between narrative and expository texts have neglected to equate important text characteristics (e.g., difficulty, cohesion) across the two text types, it is unclear whether the results of these studies are a consequence of differences between text types (narrative, expository) or differences between text characteristics that were not controlled (e.g., text difficulty, cohesion). See Appendix A for examples. Although addressing this shortcoming is not the primary focus of our study, we do begin to address it by first ensuring key characteristics are equivalent between our narrative and expository texts, and then by using total effects to determine the relative contributions that some important component skills make to comprehension of: (i) just narrative texts (i.e., within-text contributions), (ii) just expository texts (i.e., within-text contributions), and (iii) narrative versus expository texts (i.e., between-text contributions). We used total effects/SEMs instead of correlations to make comparisons among the contributions that component skills make to narrative and expository comprehension scores because total effects are based on statistics that consider all of the relationships among all of the component skills simultaneously whereas correlations are based on statistics that consider the relationship between just two component skills.

To achieve these goals, we assessed word decoding (i.e., letter-word identification, phonological decoding), text memory, knowledge integration, and working memory skills both longitudinally, when the children were in Kindergarten or early Grade 1 (i.e., time point 1) and concurrently, when the children were in Grade 2 or early Grade 3 (i.e., time point 2). Assessing these component skills at two time points afforded us the opportunity to determine whether the contributions of component skills assessed before a child begins to read (i.e., time point 1) were equivalent to the contributions of component skills assessed as a child is learning to read (i.e., time point 2). We assessed comprehension of narrative and expository texts at time point 2.

We used Coh-Metrix, a software tool that reports multiple characteristics about texts (e.g., Graesser et al., 2011), to analyze the characteristics of our texts. By analyzing the characteristics of our texts, we ensured that: (i) our narrative texts were indeed more narrative than our expository texts, and that (ii) other text characteristics (e.g., text difficulty, word frequency, number of words, number of sentences, and cohesion) were equivalent between narrative and expository texts. In addition, by equating text difficulty, word frequency, and so on, we reduced the possibility that our findings were a consequence of another text characteristic other than text type (i.e., narrativity), the only characteristic that should be different between our narrative and expository texts.

For our SEMs, we used the Construction-Integration Model (i.e., C-I Model) as our theoretical framework (e.g., Kintsch, 1998). The advantages of adopting the C-I Model are that: (i) it is considered to be the most complete and well-formed model of reading comprehension (McNamara & Magliano, 2009) and (ii) it includes word decoding and knowledge integration, two of the important component skills of reading comprehension (Garcia & Cain, 2014; Kraal et al., 2019).

**Figure 1.**
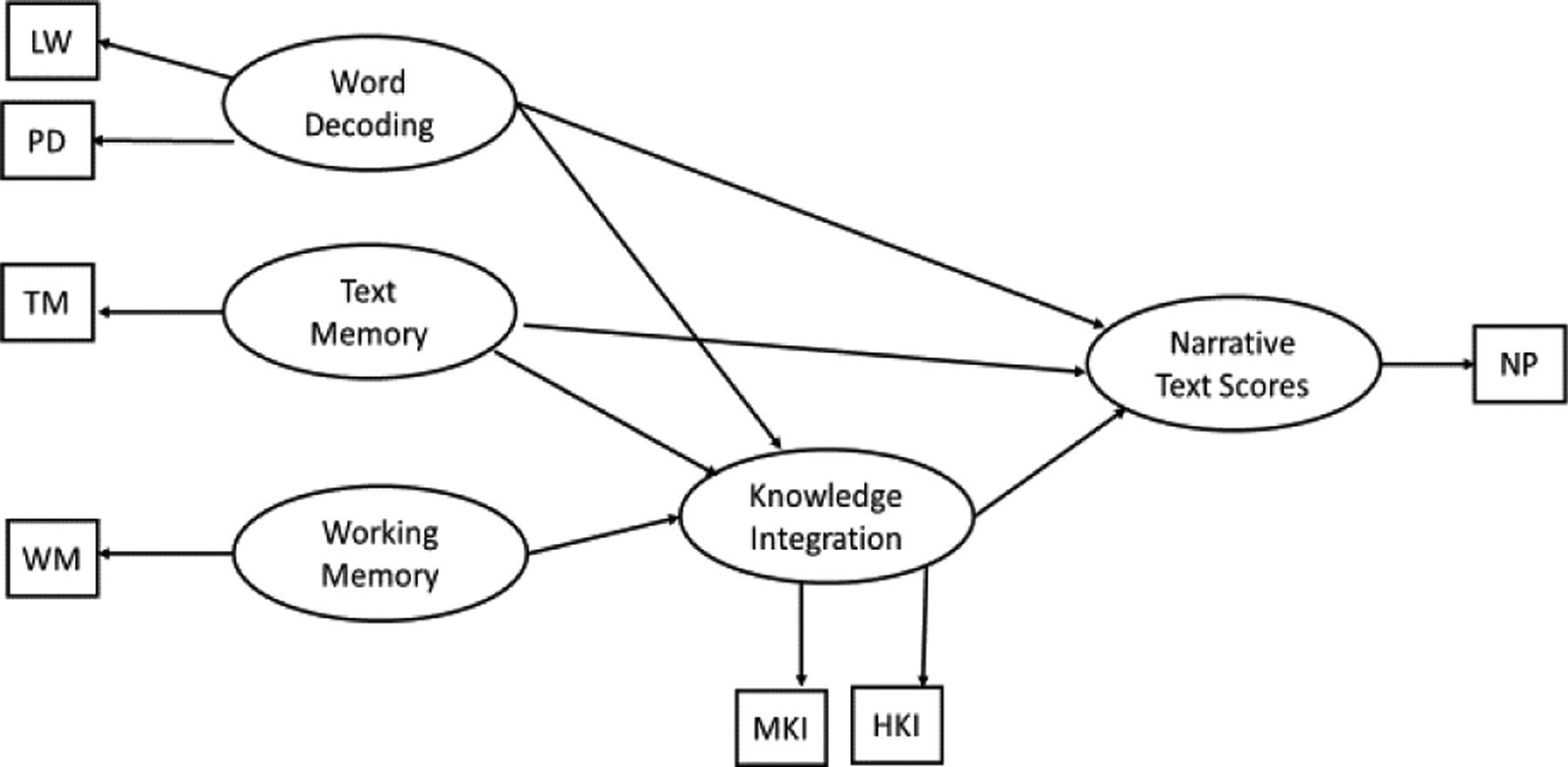
Path diagram of the situation model for narrative comprehension scores LW = letter-word identification, PD = phonological decoding, TM = text memory, WM = working memory, MKI = medium knowledge integration, HKI = high knowledge integration, NP = narrative comprehension scores.

Figure 1 depicts a hypothetical situation model SEM for narrative texts. The latent variables for the component skills and narrative texts, depicted by ovals, are measured by the observed variables. The observed variables, depicted by squares, are the scores on the actual psychometric measures for the component skills and narrative texts. Each unidirectional arrow represents a direct path and its direction of influence while the absence of a path indicates no influence. Because the situation model forms via an interplay of word decoding, text memory, and knowledge integration skills, Figure 1 depicts the latent variable for the narrative comprehension scores drawing on word decoding, text memory, and knowledge integration. Knowledge integration is particularly important in this situation model SEM because readers use knowledge integration to combine information from the text and their prior knowledge in order to form the situation model (McNamara & Magliano, 2009).

Based on previous research, we predicted that beginning readers will comprehend narrative texts better than expository texts (e.g., Best et al., 2008; Garcia & Cain, 2014). However, the relative contributions of the component skills to narrative versus expository texts are unclear because no study has assessed multiple component skills, concurrently and longitudinally, using a population of beginning readers. On the one hand, it is possible that word decoding will be more predictive of narrative texts than expository texts, a finding that replicates adult research (Best et al., 2008). On the other hand, it is possible that word decoding will make equivalent contributions to narrative and expository texts because word decoding skills are highly predictive of beginning reading (e.g., Garcia & Cain, 2014).

For knowledge integration, it is possible that knowledge integration will make greater contributions to expository texts than narrative texts, given that researchers contend that expository texts tap inferential and knowledge integration skills to a greater extent than do narrative texts (Kaakinen, Hyönä, & Keenan, 2003). On the other hand, it is possible that knowledge integration will make equivalent contributions to narrative and expository texts, especially when important text characteristics are equated between narrative and expository texts (e.g., Eason et al., 2012).

## 2. Method

### 2.1 Participants

We recruited children from South Texas elementary schools. One hundred seventy-one children completing both time points. Of these 171 children, 16 were removed from the study because they had learning difficulties (4 children), were non-native English speakers (6 children), spoke too much of another language (4 children), or were flagged as outliers in the preliminary data screening (2 children). The average age of the remaining 155 dominant English-speaking children was 5.96 years, *range* = 5.00 to 7.15 years at time point 1 (Kindergarten or early Grade 1) and 7.92 years, *range* = 6.96 to 9.12 at time point 2 (Grade 2 or early Grade 3).

There were 70 girls and 85 boys. Twenty-six of the children were of European-American descent, 117 of the children were of Hispanic descent, and the remaining 12 children were either Asian, African-American, or of mixed descent. Finally, socioeconomic status was determined by assessing household income and the highest education level of the parents. Based on the 132 completed responses, the average income was $60,000 per year, *range* = under 12,000 per year to over 100,000 per year, which is slightly above the state’s average income of $59,206. The average education level was some associates degree, which is also above the state average of having attained a high school or GED degree. All children received toy packages for participating.

### 2.2 Measures

#### 2.2.1 Word Decoding

The measures of letter-word identification and phonological decoding, administered at both time points, were the letter-word identification and word attack subtests from the Woodcock Reading Mastery Test—Revised (Woodcock, 1987). These measures have high Cronbach alphas and are suitable for ages 2 to 98. For the letter-word identification measure, children read the letters and words aloud (e.g., *m, n, dog … photograph*, etc.), and for the phonological decoding measure children reported the sounds of letters and pseudowords (e.g., *k, n, tiff, zoop,* etc.). Each measure increased in difficulty with each successive trial, and the measures were stopped after six consecutive incorrect answers.

#### 2.2.2 Text Memory and Knowledge Integration Skills

We administered measures of text memory and knowledge integration skills at both time points. These measures were subtests of Hannon and Frias’ (2012) preschooler component processes task (PR-CPT), which assesses multiple higher-level comprehension processes. Because the PR-CPT is described fully in Hannon and Frias (2012), the text memory and knowledge integration skills are only briefly described below. These descriptions are paraphrases of Hannon (2018).

The version of the PR-CPT that we administered via PowerPoint at time point 1 included five paragraphs; the first paragraph was for practice. Each paragraph included three sections: (i) an animated introduction, (ii) an aural paragraph with accompanying pictures, and (iii) aural test statements that assessed text memory and knowledge integration (Hannon, 2018). The animated introductions and aural paragraphs were presented two times before the aural test statements. The version of the PR-CPT administered at time point 2 was identical to the PR-CPT version administered at time point 1 with two exceptions. First, the animated introductions and accompanying short paragraphs were presented once rather than twice. Second, the child read the final two paragraphs rather than hearing them aurally.

In the version of the PR-CPT administered at time point 1, for each paragraph a child first listened to an animated introduction, which provided a context for a paragraph. Then, he or she listened to a two-sentence paragraph while simultaneously viewing pictures. Each two-sentence paragraph described the relations among real (e.g., *dog*) and nonsense terms (e.g., *JIMP*). After listening and viewing the animated introduction and its accompanying paragraph two times, a child answered true-false statements that assessed specific higher-level comprehension skills. The 24 *text memory* test statements assessed a child’s ability to recall information from a text. For example, the text memory statement *A JIMP looks like a DOG*. was explicitly stated in a text. The 24 *knowledge-integration* test statements assessed a child’s ability to integrate text-based information with his or her prior knowledge. These 24 knowledge integration statements consisted of two types of knowledge integration that varied in the degree they tap prior knowledge. However, for the purposes of this study, these statements were called knowledge integration, and a composite z-score was created. An example of a knowledge integration statement is *A JIMP is larger than a CAT.,* which can be deduced from the paragraph sentence *A JIMP is larger than an ELEPHANT*. and a child’s prior knowledge that *ELELPHANTS are larger than CATS*. The Cronbach alphas for the text memory and knowledge integration statements from time point 1 were .78 and .85 respectively.

#### 2.2.3 Working Memory

We administered variants of Hannon and Frias’s (2012) aural verbal working memory measure at both time points. The description here is a paraphrase of Hannon and Frias. In this measure, a child listened to a set of sentences (e.g., *He ate a bug. The family was at the park. The ducks were in the lake*.) and then at the end of the set, heard two sentences from that same set again. Each repeated sentence had a missing word, which was replaced with a beep (e.g., He ate a **beep**. The **beep** were in the lake). The child’s task was to recall the missing words (e.g., *bug, duck*). A child only received credit for generating the correct missing words from the original sentences. The set sizes for the working memory measure administered at time points 1 and 2 ranged from 2 to 5 and from 2 to 7 respectively. A child’s working memory score was based on set size (Hannon and Frias, 2012). For a child to get full credit for a set size, he or she needed to answer at least two sets correctly for the same set size (Hannon, 2018).

#### 2.2.4 Narrative and Expository Texts

We administered three narrative and three expository texts at time point 2; all six texts were age-appropriate. See Appendix A, which reports the number of narrative and expository texts that are typically used in papers. Coh-Metrix (Graesser et al., 2011), a software tool that reports multiple characteristics about texts, was used to assess narrativity and other characteristics of our two text types.

Because there are no generally accepted sets of narrative and expository texts, we selected texts from Reading for Comprehension, levels A and B (Reading for Comprehension, 2007) for two reasons. First, we observed a strong .74 correlation between comprehension accuracy scores on these narrative and expository texts and comprehension scores on the Gates-MacGinitie (MacGinitie & MacGinitie, 1989), a frequently used standardized measure of reading comprehension. Second, our narrative and expository texts had equal numbers of factual and inferential questions. This latter point is important when comparing narrative and expository texts because any observed difference in comprehension scores between these two text types cannot be attributed to narrative/expository texts having unequal numbers of factual/inferential questions.

Once the Coh-Metrix (Graesser et al., 2011) generated values for all of our reported text characteristics, we computed two-tailed t-tests to determine whether a characteristic was equivalent between narrative and expository texts. In order to reduce the chance of type 1 errors, we set a more stringent alpha (i.e., .003), which was calculated by setting a familywise alpha = .05 and then by dividing this familywise alpha by the number of t-tests (i.e., 0.05/17); see Hannon (2019) for a similar approach. Because previous studies reported few text characteristics, we included 17 text characteristics in Appendix B. The t-tests revealed that narrative texts had a significantly higher degree of narrativity than did expository texts, *M* = 86.31 versus *M* = 41.35, *t* = 4.49, *p* < .003. However, all other characteristics were equivalent between narrative and expository texts. That is, our narrative and expository texts were equivalent in: (i) difficulty (i.e., Flesch reading ease, Flesch grade level), (ii) level of cohesion (i.e., combined cohesion, situation model – temporal cohesion), (iii) number of words, (iv) number of sentences, (vi) concreteness, and (vi) word frequency (CELEX word frequency).

Each narrative and expository text consisted of two-to-four paragraphs, a small black and white picture, and five four-choice multiple-choice questions: two factual and three inferential. The two factual questions assessed information explicitly stated in a text (e.g., *Rainbows are _______*). The three inferential questions assessed information implied by a text: (i) one question queried the definition of a word, (ii) one question queried the theme of the story, and (iii) one question assessed integrating the text information with prior knowledge. Children read each text and then answered the questions. We allowed children to look back at the text while answering the questions. All unanswered questions were marked incorrect. The average percentage of missed questions was < 1.0%. The total number of correct answers on narrative texts (i.e., correct factual + correct inferential questions) and the total number of correct answers on expository texts (i.e., correct factual + correct inferential questions) were our two dependent measures. In the results, section we labeled these dependent measures as comprehension scores or scores, for short.

## 3. Results

The results consisted of three sections. Section 3.1 reported the preliminary analysis, section 3.2 determined the best predictor(s) for each text type, and section 3.3 compared the contributions of the predictors for narrative versus expository texts. To re-iterate, the word decoding skills included letter-word and phonological decoding, and the higher-level comprehension skills included text memory and knowledge integration. Narrative text comprehension scores were the total number of correct answers on multiple-choice questions on the narrative texts (i.e., number of correct factual + inferential questions). Expository text comprehension scores were the total number of correct answers on multiple-choice questions on the expository texts (i.e., number of correct factual + inferential questions).

### 3.1 Preliminary Analysis

The preliminary analysis: (i) pre-screened the data for non-linearity, lack of normality, etc., (ii) compared comprehension scores for narrative versus expository texts, (iii) briefly examined the correlations among the predictors and the two text types, and (iv) reported the amount of overall variance in comprehension scores for narrative versus expository texts. The overall variance is reported for both concurrent and longitudinal predictors.

#### 3.1.1 Pre-screening of Data

Regression analyses, that included all the predictors, generated screening statistics for our data. The screening statistics included: (i) outliers (i.e., studentized residuals), (ii) leverage data points (i.e., *h*, also known as the hat value), (iii) linearity (bivariate scatterplots), (iv) normality (i.e., normality probability plots), (v) heteroscedasity (i.e., White’s test), and (vi) multicollinearity of predictors (tolerance test). The results revealed that the data for two children had studentized residuals well beyond acceptable limits. We removed these data and repeated the regressions using the remaining data. The second analyses revealed no data abnormalities. We removed the data for these two children from all subsequent analyses.

#### 3.1.2 Differences in Comprehension Scores as a Function of Text Type

We computed a t-test to assess differences in comprehension scores for narrative versus expository texts. The results revealed that comprehension scores for narrative texts were indeed significantly higher than comprehension scores for expository texts, *t* (154) = 2.17, *p* < .04, a finding that is consistent with previous research (e.g., Best et al., 2008; Diakidoy et al., 2005).

#### 3.1.3 Correlational and Regression Analyses

Tables 1 and 2 report the correlations among scores for the longitudinal and concurrent predictors respectively. As both tables show, all predictors significantly correlated with narrative and expository text scores. In addition, the regression analyses, reported by LISREL, indicated that the concurrent predictors accounted for 82.1% of the variance in narrative text scores and 59.5% of the variance in expository text scores while the longitudinal predictors accounted for 38.1% of the variance in narrative text scores and 26.2% of the variance in expository text scores.

### 3.2. Best Predictor(s) for Each Text Type

We used total effects to determine the best predictor(s) (i.e., component skills) for scores on each text type. We also used total effects to determine the relative powers of a component skill to predict narrative versus expository text scores. These total effects were computed from direct and indirect effects reported by the SEMs. The SEMs were created using LISREL 9.10 (Jöreskog & Sörbom, 2013) and maximum likelihood estimation.

There were four SEMs: Two narrative SEMs, one with concurrent predictors and one with longitudinal predictors, and two expository SEMs, one with concurrent predictors and one with longitudinal predictors. In each SEM, a latent variable was considered to exert influence on another latent variable when the path coefficient between the latent variables was significantly different from 0.00 (Hannon & Daneman, 2014, p. 183). Statistical significance of a path was determined using two-tailed t-tests with a *p* < .05. Solid lines in figures represent statistically significant paths, and dashed lines represent non-significant paths (Hannon, 2016). We assessed SEM fits using a collection of fit statistics, including the Goodness-of-fit Index (GFI), the Comparative Fit Index (CFI), and the Root Mean Square Error of Approximation (RMSEA). Table 3 reports the fit statistics for the SEMs, and Table 4 reports the direct, indirect, and total effects for concurrent and longitudinal predictors as a function of text type.

In order to identify a significant difference between two predictors, we followed a procedure proposed by Schermelleh–Engel (2021); see also Klopp (2020). First, we created two additional SEMs: (i) a SEM with an equality constraint between the two paths of interest (i.e., predictor path 1 = predictor path 2), and (ii) a SEM that did not have this constraint (i.e., the two predictor paths were free to calculate). Then, we computed a *χ2-difference* test between the constrained and unconstrained SEMs. If the *χ2-difference* test was significant, *p* < .05, we concluded that the magnitudes of the two predictors differed significantly.

**Figure 2.**
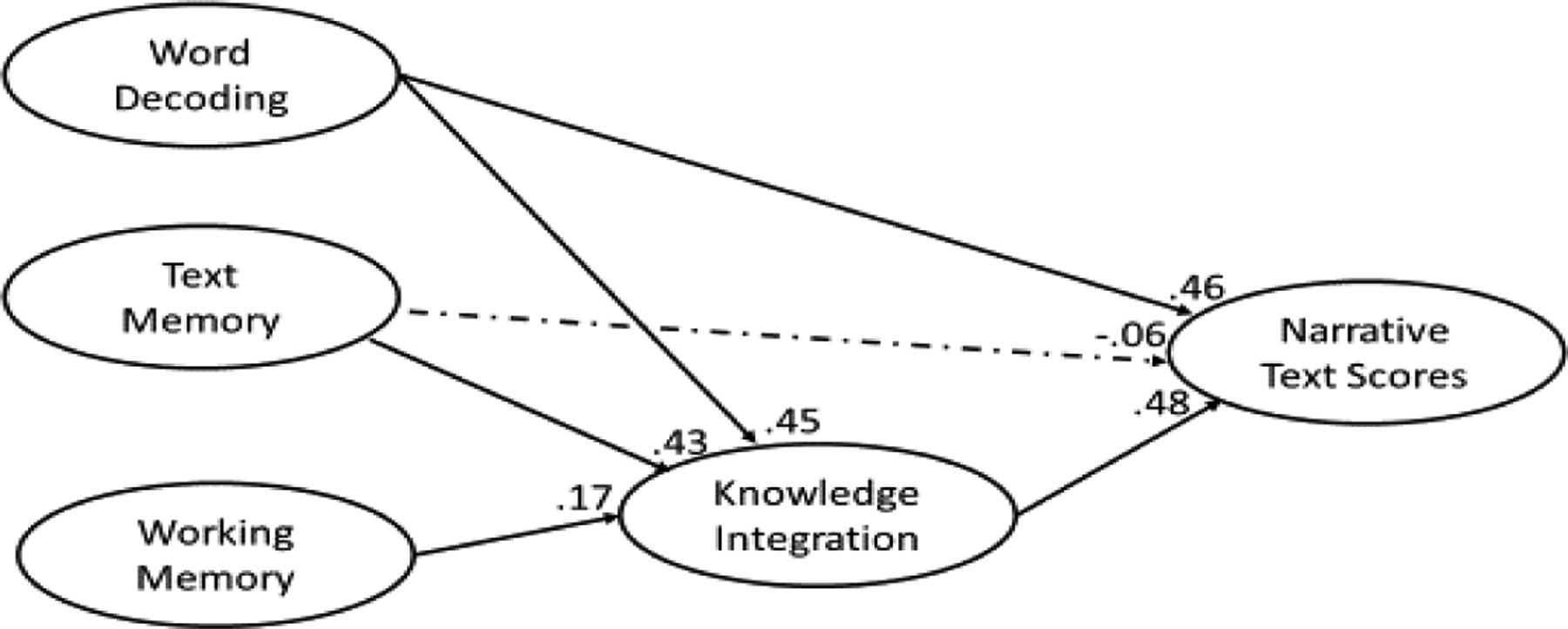
Situation model SEM for narrative comprehension scores using concurrent predictors

**Figure 3.**
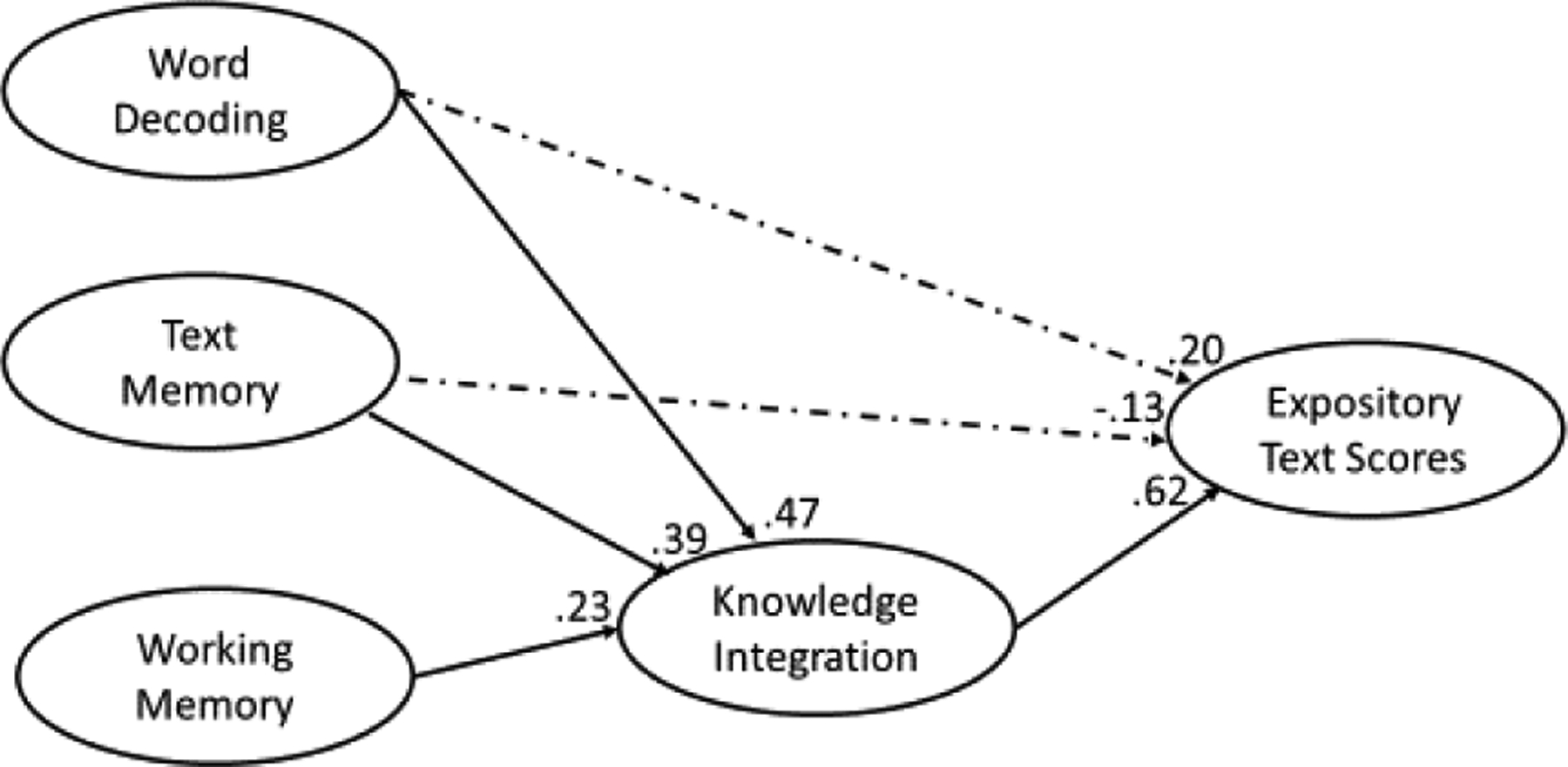
Situation model SEM for expository comprehension scores using concurrent predictors

As Table 3 shows, all fit statistics for the SEMs were well within acceptable limits; a finding that suggests the SEMs for narrative and expository text scores were suitable for explaining the data. Moreover, as Figures 2 and 3 show, nearly all the paths leading from the predictors to narrative and expository texts were significant with three exceptions. The first exception was the presence of a non-significant path leading from text memory to narrative texts scores (depicted as dashed lines) in the narrative SEM; a finding that suggests that the influence text memory exerts on narrative text scores is not direct but rather indirect (i.e., from text memory → knowledge integration → narrative scores). The other two exceptions were the non-significant paths leading from text memory to expository text scores and from word decoding to expository text scores (depicted as dashed lines) in the expository SEM; a finding that suggests that the influences text memory and word decoding exert on expository text scores are not direct but rather indirect (from text memory → knowledge integration → expository scores; word decoding → knowledge integration → expository scores).

#### 3.2.1 Best Predictor(s) for Narrative Texts

Table 4 depicts the direct and indirect effects of the predictors on narrative and expository text scores as a function of the time the predictors were administered. To re-iterate, concurrent predictors were administered at the same time as the narrative and expository texts were assessed. In the present study this assessment happened when the children were in Grade 2 (i.e., time point 2). Longitudinal predictors were administered prior to the administration of the narrative and expository texts. In the present study this assessment happened when the children were in Kindergarten (i.e., time point 1).

As Table 4 shows, when word decoding was assessed concurrently with narrative and expository texts, it exerted more influence on narrative text scores than either knowledge integration, text memory, or working memory, .68 versus .48, .15 and .08 respectively, minimum *χ*^*2*^*-difference* (1) = 51.26, *p* < .05. Moreover, knowledge integration exerted more influence on narrative text scores than either text memory or working memory, .48 versus .15 and .08 respectively, minimum *χ*^*2*^*-difference* (1) = 54.66, *p* < .05. On the other hand, when word decoding was assessed longitudinally, it exerted the same influence as knowledge integration, .40 and .35 respectively, *χ*^*2*^*-difference* (1) = 2.37, *p* > .05. The remaining two longitudinal predictors, text memory and working memory, exerted considerably less influence, .14 and .15 respectively.

#### 3.2.2 Best Predictor(s) for Expository Texts

As Table 4 shows, knowledge integration exerted more influence on expository text scores than any other concurrent or longitudinal predictor: Concurrently (i.e., .62 versus .49, .11, and .14) and longitudinally (i.e., .40 versus .24, .13, and .14), minimum *χ*^*2*^*-difference* (1) = 45.01, *p* < .05. This finding is inconsistent with the assumption that word decoding is the most important predictor for beginning reading comprehension (e.g., Garcia & Cain, 2014).

### 3.3 Comparisons of Predictors between Narrative and Expository Texts

So far, our results reveal that the relative influences that predictors exert on just narrative texts are different from the relative influences that those same predictors exert on just expository texts. However, it is also plausible that the influence exerted by a predictor also varies between text types. For instance, perhaps word decoding exerts more influence on narrative text scores than expository text scores.

**Figure 4.**
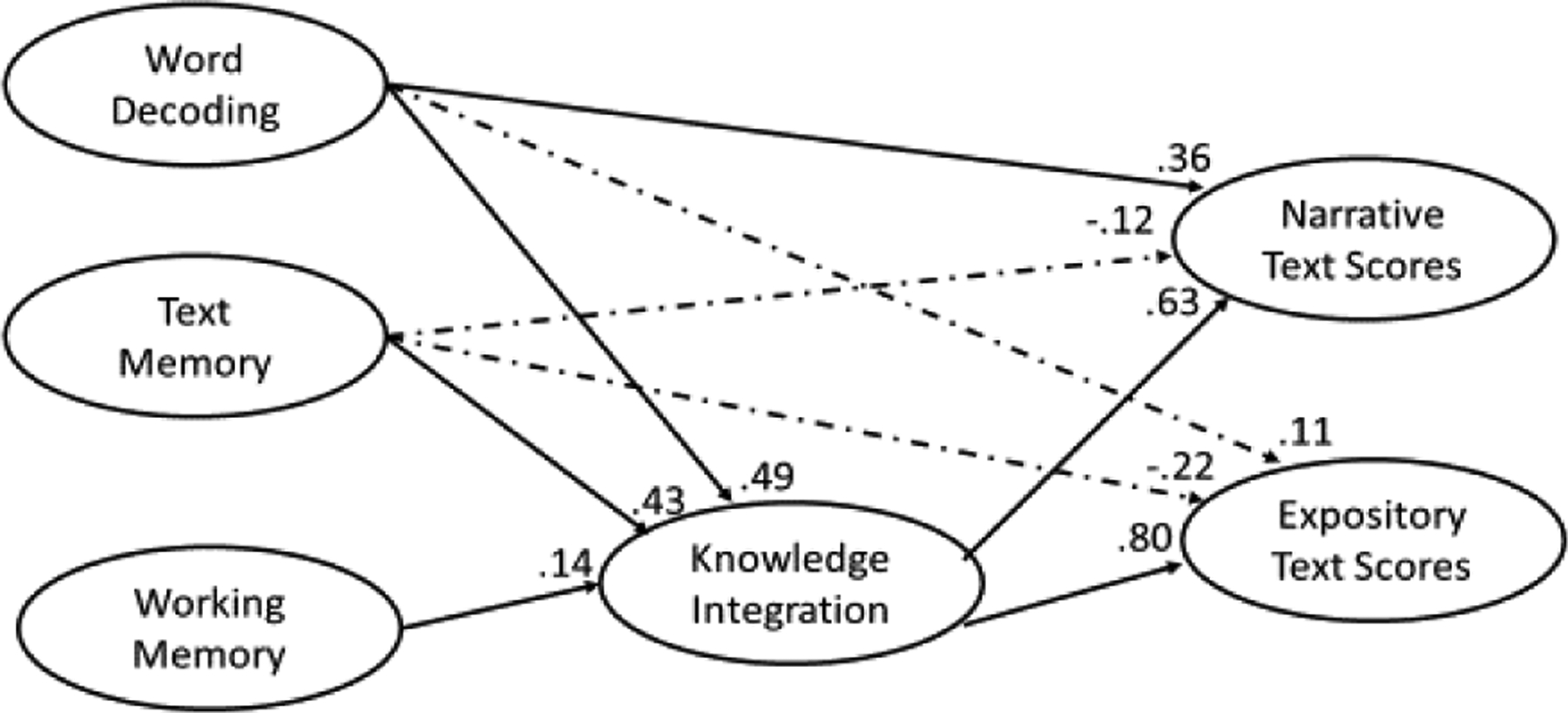
Situation model SEM for narrative and expository comprehension scores using concurrent predictors

To test for this possibility, we created new SEMs to compute the total effects of word decoding, text memory, knowledge integration, and working memory for narrative versus expository text scores. These SEMs were identical to the earlier situation model SEMs, except they included narrative and expository text scores in the same SEM. Table 3 reports the fit statistics for the concurrent and longitudinal combined SEMs, and Figure 4 depicts the SEM that included concurrent predictors.

As Table 3 shows, the combined SEM using concurrent predictors fit the data well; however, the combined SEM using longitudinal predictors had a poorer fit for the data, *RMSEA* = .18. More interestingly, as Table 5 shows, the total effects for the concurrent predictors varied greatly between the two text types. In particular, word decoding exerted more influence on narrative text scores than expository text scores, .67 versus .50, χ^2^ (1) = 68.59, *p* < .05, whereas knowledge integration exerted more influence on expository text scores than narrative text scores, .80 versus .63 respectively, χ^2^ (1) = 12.58, *p* < .05. The remaining two predictors, text memory and working memory, exerted equivalent influences on narrative and expository text scores: Text memory, .15 versus .12 respectively and working memory, .09 versus .11 respectively. In addition, as Table 5 shows, the pattern of total effects for longitudinal predictors was identical to the pattern observed for the concurrent predictors; although, the differences in effect sizes were not as profound. That is, word decoding exerted more influence on narrative text scores than expository text scores, .38 versus .23, χ^2^ (1) = 68.59, *p* < .05, whereas knowledge integration exerted more influence on expository text scores than narrative text scores, .61 versus .55 respectively, χ^2^ (1) = 8.07, *p* < .05. The remaining two predictors, text memory and working memory, exerted equivalent influences on narrative and expository text scores: Text memory, .14 versus .14 respectively and working memory, .20 versus .23 respectively.

## 4. Discussion

The present study examined the relative contributions that word decoding, text memory, knowledge integration, and working memory make to: (i) narrative text scores, (ii) expository text scores, and (iii) narrative versus expository text scores. The results revealed that although word decoding was the best concurrent predictor of narrative text scores, word decoding was not the best predictor of expository text scores. Moreover, although word decoding was a better predictor of narrative text scores than expository text scores, knowledge integration was a better predictor of expository text scores than narrative text scores. Below, we discuss these results in more detail.

### 4.1 Predictors of Narrative Comprehension Scores

The results only partially supported the common assumption that word decoding is the best predictor of early reading (e.g., Garcia & Cain, 2014). In particular, our results showed that word decoding was the best concurrent predictor of narrative text scores, but word decoding and knowledge integration were equivalent longitudinal predictors. Given that word decoding skills vary greatly among Kindergarten, it is possible this variability is reduced between Kindergarten and Grade 2 because of the extensive practice word decoding skills receive in early elementary school settings.

### 4.2 Predictors of Expository Comprehension Scores

Our results did not support the common assumption that word decoding is the best predictor of early reading (e.g., Garcia & Cain, 2014). Rather, our results showed that knowledge integration was the best predictor of expository text scores, both concurrently and longitudinally. Given that research examining the comprehension of expository texts in the earlier grades of elementary school (i.e., Grades 1 and 2) is scarce (Kraal et al., 2017), it is possible that the assumption that word decoding is the best predictor of early reading was based largely on studies that assessed comprehension of just narrative texts. Future research may wish to examine this possibility.

### 4.3 Comparisons of Predictors between Text Types

Consistent with previous adult and child research (e.g., Best et al., 2008; Diakidoy et al., 2005; Kraal et al., 2017; Kraal et al., 2019), comprehension scores were significantly higher on narrative than expository texts. Also, consistent with previous research (e.g., Best et al., 2008), word decoding was more predictive of comprehension scores on narrative texts than expository texts. More interestingly, knowledge integration was more predictive of comprehension scores on expository texts than narrative texts, an important novel finding. From a practical perspective, these findings highlight the importance of text genre when determining which component skills are most important for early reading comprehension. In particular, word decoding is more central to narrative text scores, whereas knowledge integration is more central to expository text scores. But even more importantly, these results call into question the common assumption that word decoding is the most important component skill for beginning readers. This finding is important for educators because it suggests that word decoding is not the only component skill that should be emphasized during early literacy training. Rather, there is a need to emphasize both knowledge integration skills and perhaps even expository texts.

But why does knowledge integration predict expository texts better than narrative texts? One possibility is that narrative texts provide less opportunity to integrate prior knowledge with passage information than do expository texts. By this account because narrative texts are often fictitious, there is less opportunity/need for readers to use their knowledge integration skills to incorporate prior knowledge into the situation model of the text. In contrast, because expository texts are often factual, there is more opportunity/need for readers to use their knowledge integration skills to incorporate prior knowledge into the situation model of the text.

### 4.4 Limitations

The present study also has limitations that future research should address. First, although our findings complement Best et al. (2008), who showed that world or prior knowledge is more predictive of expository text scores than narrative text scores, we did not assess background knowledge in our study because we wanted to limit our focus to cognitive skills. However, because of this choice, we do not know the overlapping or unique contributions that background knowledge and knowledge integration make to comprehension scores. Second, the present study targeted a young age group because we know little about how component skills exert influences, both concurrently and longitudinally, on narrative versus expository texts for this age group. However, we urge caution when generalizing the present findings to other populations because younger children may use different component skills to different extents than do older children or adults. Finally, although the present study used more narrative and expository texts than a number of other studies (see Appendix A), future research should use a larger number of texts in order to verify the present results generalize to a larger number of texts. In addition, perhaps future research could include other measures assessing comprehension of narrative and expository texts, such as free recall.

### 4.5 Summary and Conclusions

The present study examined the relative contributions that word decoding, text memory, knowledge integration, and working memory made to: (i) narrative text scores, (ii) expository text scores, and (iii) narrative versus expository text scores. The results revealed that although word decoding was the best concurrent predictor of narrative text scores, word decoding was not the best predictor of expository text scores. Rather, knowledge integration was the best predictor of expository scores. Moreover, although word decoding was a better predictor of narrative text scores than expository text scores, knowledge integration was a better predictor of expository text scores than narrative text scores. These findings are important for educators who seek to customize reading interventions for specific types of readers.

## Figures and Tables

**Table 1.** Correlations among Scores for Narrative Texts, Expository Texts, and Longitudinal Predictors (*n* = 155)

	1	2	3	4	5	6	7
1. Narrative texts	- - -	.63*	.48*	.39*	.33*	.44*	.29*
2. Expository texts		- - -	.34*	.27*	.29*	.41*	.24*
3. Letter-word identification			- - -	.86*	.35*	.46*	.29*
4. Phonological decoding				- - -	.33*	.47*	.20*
5. Text-based memory					- - -	.60*	.37*
6. Knowledge integration						- - -	.55*
7. Working memory							- - -
Mean	11.68	11.26	20.65	5.15	17.57	0.01	4.05
Standard deviation	3.01	2.50	8.74	4.35	3.15	1.76	0.99

Note.

* *p* < .05. Longitudinal predictors were assessed at time point 1, narrative and expository texts were assessed at time point 2. Letter-word identification and phonological decoding were both classified as word decoding skills.

**Table 2.** Correlations among Scores for Narrative Texts, Expository Texts and Concurrent Predictors (*n* = 155)

	1	2	3	4	5	6	7
1. Narrative texts	- - -	.63*	.72*	.65*	.45*	.53*	.21*
2. Expository texts		- - -	.56*	.46*	.33*	.45*	.25*
3. Letter-word identification			- - -	.83*	.37*	.42*	.20*
4. Phonological decoding				- - -	.36*	.42*	.18*
5. Text memory					- - -	.60*	.26*
6. Knowledge integration						- - -	.31*
7. Working memory							- - -
Mean	11.68	11.26	43.26	16.76	19.92	0.02	5.34
Standard deviation	3.01	2.50	7.90	6.73	2.58	1.55	1.01

Note.

* *p* < .05. Concurrent predictors, narrative texts and expository texts were all assessed at time point 2. Letter-word identification and phonological decoding were both classified as word decoding skills.

**Table 3.** Fit Statistics for Narrative, Expository and Combined SEMs

Models	χ^2^	*df*	*p-exact*	*p-close*	*GFI*	*RMSEA*	*CFI*
(i) Narrative – Situation Model SEMs							
Concurrent predictors	6.89	7	0.4407	0.68	.99	.00	1.00
Longitudinal predictors	13.95	7	0.0521	0.18	.98	.08	0.99
(ii) Expository – Situation Models SEMs							
Concurrent predictors	5.92	7	0.5493	0.76	.99	.00	1.00
Longitudinal predictors	14.01	7	0.0510	0.18	.98	.08	0.99
(iii) Combined – Situation Model SEMs							
Concurrent situation model	10.94	11	0.4486	0.74	.98	.00	1.00
Longitudinal situation model	58.39	11	0.0000	0.00	.91	.18	0.94

***Note*.** Combined SEMs include both narrative and expository text comprehension scores.

**Table 4.** Direct, Indirect, and Total Effects of Concurrent and Longitudinal Predictors for Just Narrative Text Scores and Just Expository Text Scores

	Narrative			Expository .		
	Direct	Indirect	Total	Direct	Indirect	Total
(i) Concurrent Predictors						
Knowledge integration	.48	---	.48	.62	---	.62
Word decoding	.46	.22	.68	.20^a^	.29	.49
Text memory	*−.06* ^a^	.21	.15	*−.13* ^a^	.24	.11
Working memory	---	.08	.08	---	.14	.14
(ii) Longitudinal Predictors						
Knowledge integration	.35	---	.35	.40	---	.40
Word decoding	.28	.12	.40	*.10* ^a^	.14	.24
Text memory	*.01* ^a^	.13	.14	*−.01* ^a^	.14	.13
Working memory	---	.15	.15	---	.14	.14

**Note**.

a = non-significant path. Total = direct + indirect. All effects taken from situation model SEMs assessing a single text type. For example, narrative situation model SEM with concurrent predictors. See Figures 2 and 3 for narrative and expository SEMs with concurrent predictors.

**Table 5.** Direct, Indirect, and Total Influences of Concurrent and Longitudinal Predictors for Narrative versus Expository Text Scores taken from Combined Situation Model SEMs

	Narrative			Expository .		
	Direct	Indirect	Total	Direct	Indirect	Total
(i) Concurrent Predictors						
Knowledge integration	.63	---	.63	.80	---	.80
Word decoding	.36	.31	.67	*.11* ^a^	.39	.50
Text memory	*−.12* ^a^	.27	.15	*−.22* ^a^	.34	.12
Working memory	---	.09	.09	---	.11	.11
(ii) Longitudinal Predictors						
Knowledge integration	.55	---	.55	.61	---	.61
Word decoding	.19	.19	.38	*.02* ^a^	.21	.23
Text memory	*−.08* ^a^	.22	.14	*−.10* ^a^	.24	.14
Working memory	---	.20	.20	---	.23	.23

Note.

a = non-significant path. Total = direct + indirect. All influences taken from combined situation model SEMs that assessed narrative and expository text scores simultaneously. See Figure 4 for the SEM.

## Appendix A: Text Characteristics and Number of Texts and Questions in Sample of Previous Research Studies

**Table:**

	Narrativity Assessed	Number of Texts		Number of Factual Quest		Number of Inferential Quest	
		Nar	Exp	Nar	Exp	Nar	Exp
Population – adults							
Karlsson et al. (2018)	no	2	2	0	0	0	0
Population - children							
Best et al. (2008)	yes	1	1	6	6	6	6
Diakidoy et al. (2005)	no	1	1	7	7	7	7
Eason et al – grade 4 only	yes	?	?	5	11	9	5
Kraal et al. (2017)	no	2	2	?	?	?	?
Wu et al. (2020).	yes	1	1	?	?	?	?
Present Study	yes	3	3	6	9	6	9

*Note*. ? = information not provided.

## Appendix B: Summary of Analyses of Coh-Metrix Indices as a Function of Text Type

**Table:**

Coh-Metrix Index	Narrative Mean(std)	Expository Mean(std)
Number of sentences	16.67(6.81)	13.33(5.86)
Number of words	128.67(51.69)	99.33(44.79)
Avg number of words per sentence	7.74(0.64)	7.56(0.34)
Avg number of syllables per word	1.21(0.07)	1.22(0.01)
Narrativity	86.31(5.67)	41.35(12.97)
Combined cohesion^1^	70.58(3.40)	70.18(9.35)
LSA adjacent sentence overlap	0.24(0.07)	0.42(0.09)
LSA overlap sentences in paragraph	0.25(0.23)	0.37(0.10)
LSA overlap adjacent paragraph	0.42(0.23)	0.62(0.11)
CELEX word frequency	2.64(0.06)	2.39(0.06)
Concrete content word	401.87 (42.11)	424.58(28.46)
Flesch reading ease	95.90(4.94)	96.41(1.15)
Flesch grade level	1.65(1.04)	1.69(0.08)
Situation model – causal verb	67.05(20.56)	73.26(35.85)
Situation model – intention verb	44.35(49.56)	49.44(12.74)
Situation model - LSA verb overlap	0.05(0.01)	0.13(0.09)
Situation model – temporal cohesion	0.93(0.01)	0.87(0.07)

Note.

1 Combined cohesion is a combination of Coh-Metrix measures.
